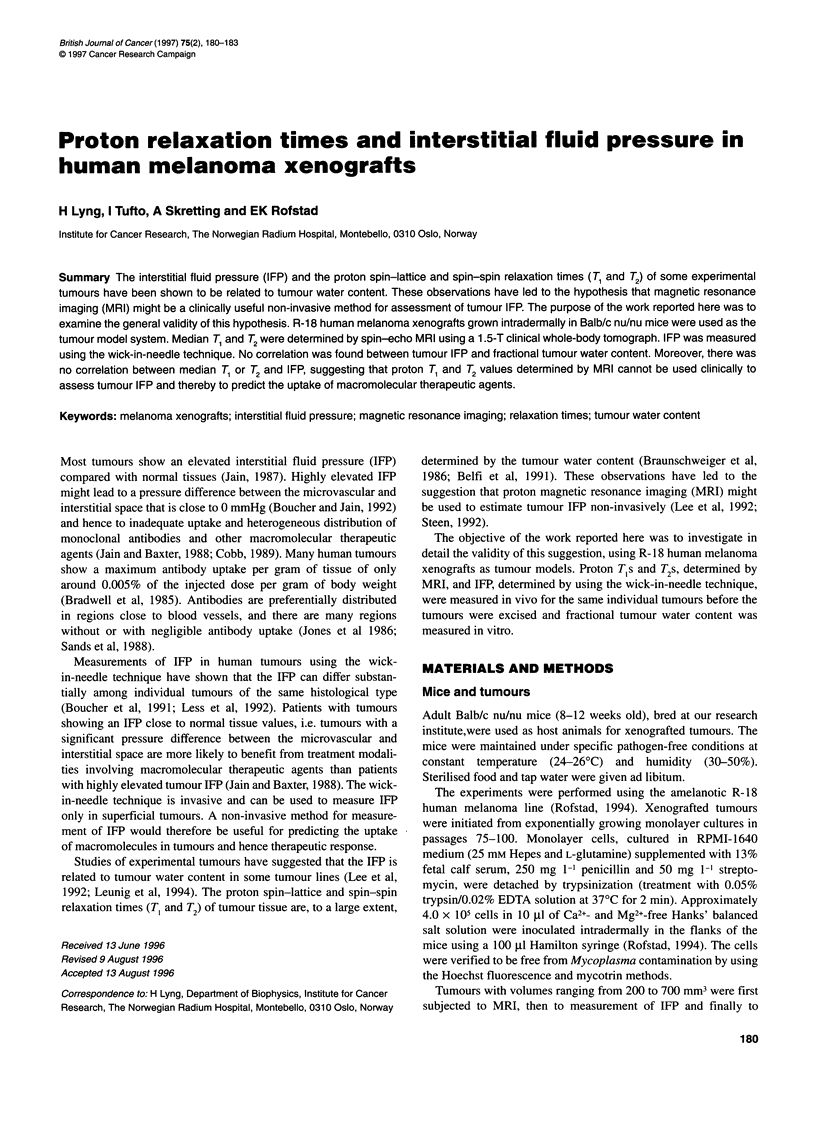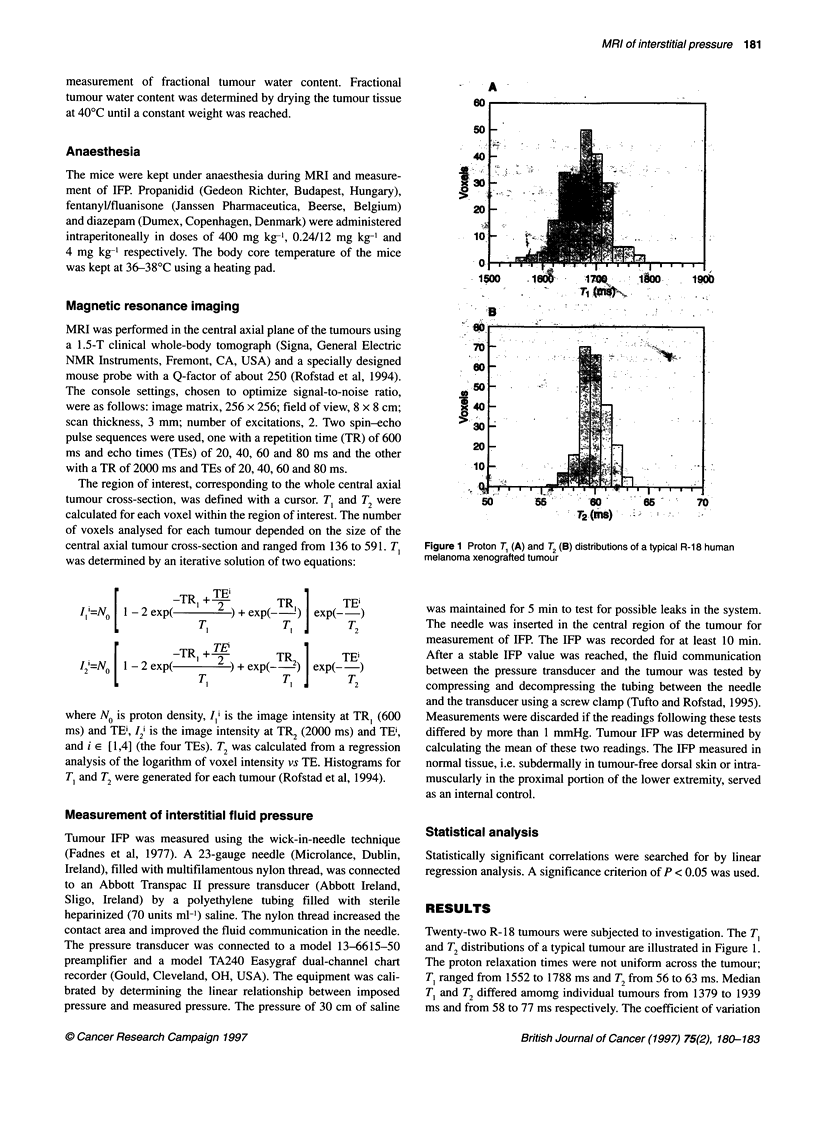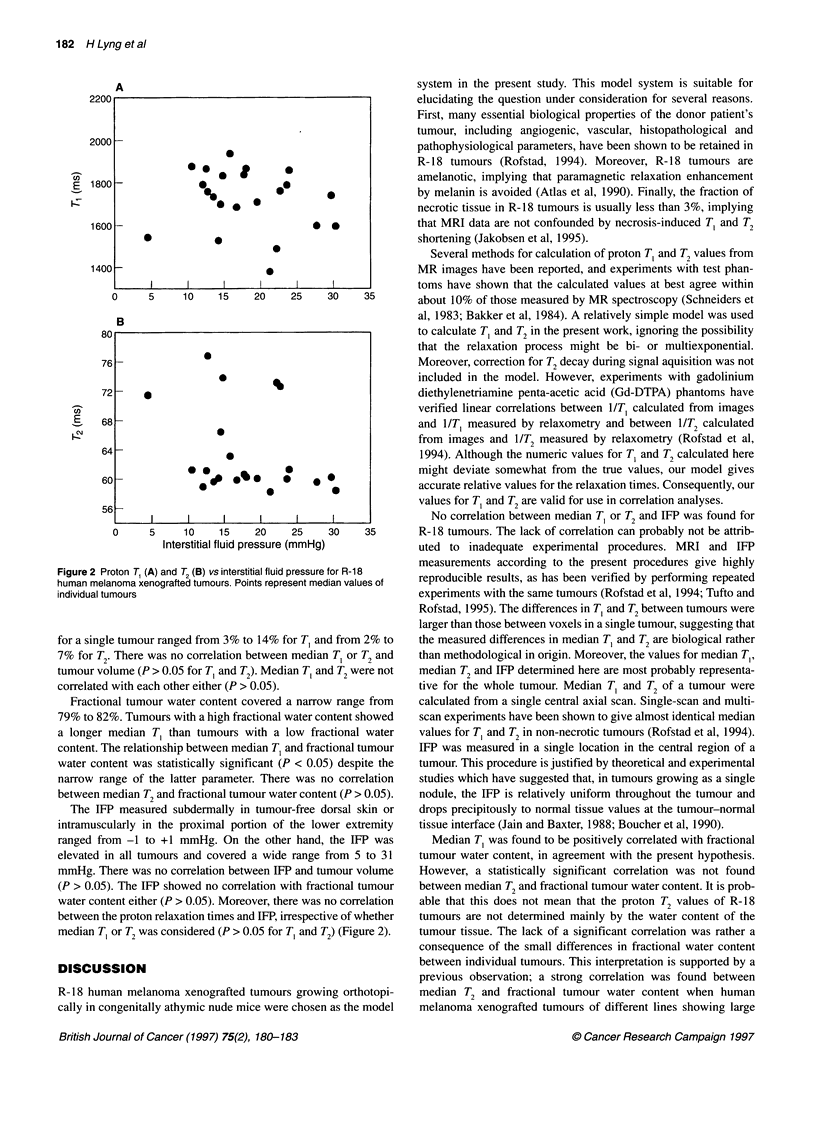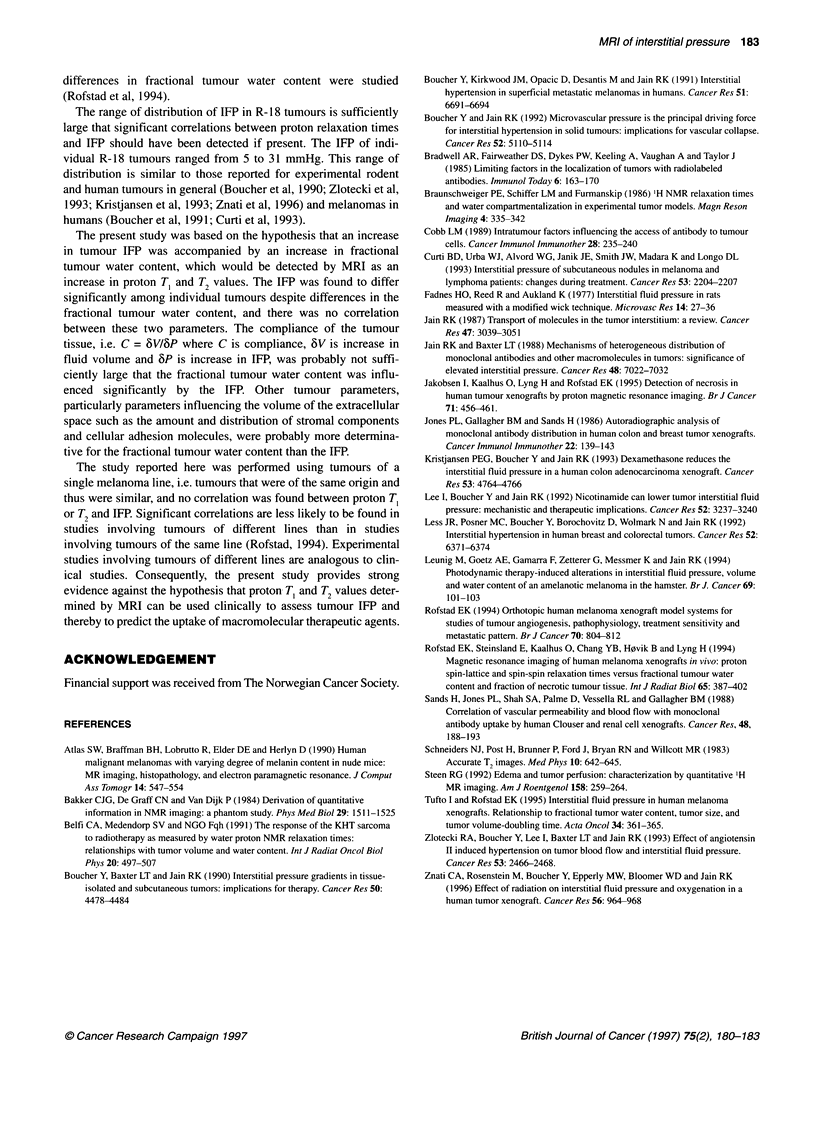# Proton relaxation times and interstitial fluid pressure in human melanoma xenografts.

**DOI:** 10.1038/bjc.1997.30

**Published:** 1997

**Authors:** H. Lyng, I. Tufto, A. Skretting, E. K. Rofstad

**Affiliations:** Institute for Cancer Research, Norwegian Radium Hospital, Montebello, Oslo, Norway.

## Abstract

The interstitial fluid pressure (IFP) and the proton spin-lattice and spin-spin relaxation times (T1 and T2) of some experimental tumours have been shown to be related to tumour water content. These observations have led to the hypothesis that magnetic resonance imaging (MRI) might be a clinically useful non-invasive method for assessment of tumour IFP. The purpose of the work reported here was to examine the general validity of this hypothesis. R-18 human melanoma xenografts grown intradermally in Balb/c nu/nu mice were used as the tumour model system. Median T1 and T2 were determined by spin-echo MRI using a 1.5-T clinical whole-body tomograph. IFP was measured using the wick-in-needle technique. No correlation was found between tumour IFP and fractional tumour water content. Moreover, there was no correlation between median T1 or T2 and IFP, suggesting that proton T1 and T2 values determined by MRI cannot be used clinically to assess tumour IFP and thereby to predict the uptake of macromolecular therapeutic agents.


British Journal of Cancer (1997) 75(2), 180–183
© 1997 Cancer Research Campaign

### Proton relaxation times and interstitial fluid pressure in human melanoma xenografts

**H Lyng, I Tufto, A Skretting and EK Rofstad**

Institute for Cancer Research, The Norwegian Radium Hospital, Montebello, 0310 Oslo, Norway

**Summary** The interstitial fluid pressure (IFP) and the proton spin–lattice and spin–spin relaxation times ($T_1$ and $T_2$) of some experimental tumours have been shown to be related to tumour water content. These observations have led to the hypothesis that magnetic resonance imaging (MRI) might be a clinically useful non-invasive method for assessment of tumour IFP. The purpose of the work reported here was to examine the general validity of this hypothesis. R-18 human melanoma xenografts grown intradermally in Balb/c nu/nu mice were used as the tumour model system. Median $T_1$ and $T_2$ were determined by spin–echo MRI using a 1.5-T clinical whole-body tomograph. IFP was measured using the wick-in-needle technique. No correlation was found between tumour IFP and fractional tumour water content. Moreover, there was no correlation between median $T_1$ or $T_2$ and IFP, suggesting that proton $T_1$ and $T_2$ values determined by MRI cannot be used clinically to assess tumour IFP and thereby to predict the uptake of macromolecular therapeutic agents.

**Keywords:** melanoma xenografts; interstitial fluid pressure; magnetic resonance imaging; relaxation times; tumour water content

Most tumours show an elevated interstitial fluid pressure (IFP) compared with normal tissues (Jain, 1987). Highly elevated IFP might lead to a pressure difference between the microvascular and interstitial space that is close to 0 mmHg (Boucher and Jain, 1992) and hence to inadequate uptake and heterogeneous distribution of monoclonal antibodies and other macromolecular therapeutic agents (Jain and Baxter, 1988; Cobb, 1989). Many human tumours show a maximum antibody uptake per gram of tissue of only around 0.005% of the injected dose per gram of body weight (Bradwell et al, 1985). Antibodies are preferentially distributed in regions close to blood vessels, and there are many regions without or with negligible antibody uptake (Jones et al 1986; Sands et al, 1988).

Measurements of IFP in human tumours using the wick-in-needle technique have shown that the IFP can differ substantially among individual tumours of the same histological type (Boucher et al, 1991; Less et al, 1992). Patients with tumours showing an IFP close to normal tissue values, i.e. tumours with a significant pressure difference between the microvascular and interstitial space are more likely to benefit from treatment modalities involving macromolecular therapeutic agents than patients with highly elevated tumour IFP (Jain and Baxter, 1988). The wick-in-needle technique is invasive and can be used to measure IFP only in superficial tumours. A non-invasive method for measurement of IFP would therefore be useful for predicting the uptake of macromolecules in tumours and hence therapeutic response.

Studies of experimental tumours have suggested that the IFP is related to tumour water content in some tumour lines (Lee et al, 1992; Leunig et al, 1994). The proton spin–lattice and spin–spin relaxation times ($T_1$ and $T_2$) of tumour tissue are, to a large extent, determined by the tumour water content (Braunschweiger et al, 1986; Belfi et al, 1991). These observations have led to the suggestion that proton magnetic resonance imaging (MRI) might be used to estimate tumour IFP non-invasively (Lee et al, 1992; Steen, 1992).

The objective of the work reported here was to investigate in detail the validity of this suggestion, using R-18 human melanoma xenografts as tumour models. Proton $T_1$s and $T_2$s, determined by MRI, and IFP, determined by using the wick-in-needle technique, were measured in vivo for the same individual tumours before the tumours were excised and fractional tumour water content was measured in vitro.

*Received 13 June 1996*
*Revised 9 August 1996*
*Accepted 13 August 1996*

*Correspondence to:* H Lyng, Department of Biophysics, Institute for Cancer Research, The Norwegian Radium Hospital, Montebello, 0310 Oslo, Norway

### MATERIALS AND METHODS

### Mice and tumours

Adult Balb/c nu/nu mice (8–12 weeks old), bred at our research institute,were used as host animals for xenografted tumours. The mice were maintained under specific pathogen-free conditions at constant temperature (24–26°C) and humidity (30–50%). Sterilised food and tap water were given ad libitum.

The experiments were performed using the amelanotic R-18 human melanoma line (Rofstad, 1994). Xenografted tumours were initiated from exponentially growing monolayer cultures in passages 75–100. Monolayer cells, cultured in RPMI-1640 medium (25 mM Hepes and L-glutamine) supplemented with 13% fetal calf serum, 250 mg $l^{-1}$ penicillin and 50 mg $l^{-1}$ streptomycin, were detached by trypsinization (treatment with 0.05% trypsin/0.02% EDTA solution at 37°C for 2 min). Approximately $4.0 \times 10^5$ cells in 10 µl of $Ca^{2+}$- and $Mg^{2+}$-free Hanks' balanced salt solution were inoculated intradermally in the flanks of the mice using a 100 µl Hamilton syringe (Rofstad, 1994). The cells were verified to be free from *Mycoplasma* contamination by using the Hoechst fluorescence and mycotrin methods.

Tumours with volumes ranging from 200 to 700 $mm^3$ were first subjected to MRI, then to measurement of IFP and finally to measurement of fractional tumour water content. Fractional tumour water content was determined by drying the tumour tissue at 40°C until a constant weight was reached.

### Anaesthesia

The mice were kept under anaesthesia during MRI and measurement of IFP. Propanidid (Gedeon Richter, Budapest, Hungary), fentanyl/fluanisone (Janssen Pharmaceutica, Beerse, Belgium) and diazepam (Dumex, Copenhagen, Denmark) were administered intraperitoneally in doses of 400 mg $kg^{-1}$, 0.24/12 mg $kg^{-1}$ and 4 mg $kg^{-1}$ respectively. The body core temperature of the mice was kept at 36–38°C using a heating pad.

### Magnetic resonance imaging

MRI was performed in the central axial plane of the tumours using a 1.5-T clinical whole-body tomograph (Signa, General Electric NMR Instruments, Fremont, CA, USA) and a specially designed mouse probe with a Q-factor of about 250 (Rofstad et al, 1994). The console settings, chosen to optimize signal-to-noise ratio, were as follows: image matrix, 256 × 256; field of view, 8 × 8 cm; scan thickness, 3 mm; number of excitations, 2. Two spin–echo pulse sequences were used, one with a repetition time (TR) of 600 ms and echo times (TEs) of 20, 40, 60 and 80 ms and the other with a TR of 2000 ms and TEs of 20, 40, 60 and 80 ms.

The region of interest, corresponding to the whole central axial tumour cross-section, was defined with a cursor. $T_1$ and $T_2$ were calculated for each voxel within the region of interest. The number of voxels analysed for each tumour depended on the size of the central axial tumour cross-section and ranged from 136 to 591. $T_1$ was determined by an iterative solution of two equations:

$$I_1^i = N_0 \left[ 1 - 2\exp\left(\frac{-TR_1 + \frac{TE^i}{2}}{T_1}\right) + \exp\left(-\frac{TR_1}{T_1}\right) \right] \exp\left(-\frac{TE^i}{T_2}\right)$$

$$I_2^i = N_0 \left[ 1 - 2\exp\left(\frac{-TR_1 + \frac{TE^i}{2}}{T_1}\right) + \exp\left(-\frac{TR_2}{T_1}\right) \right] \exp\left(-\frac{TE^i}{T_2}\right)$$

where $N_0$ is proton density, $I_1^i$ is the image intensity at $TR_1$ (600 ms) and $TE^i$, $I_2^i$ is the image intensity at $TR_2$ (2000 ms) and $TE^i$, and $i \in [1,4]$ (the four TEs). $T_2$ was calculated from a regression analysis of the logarithm of voxel intensity *vs* TE. Histograms for $T_1$ and $T_2$ were generated for each tumour (Rofstad et al, 1994).

### Measurement of interstitial fluid pressure

Tumour IFP was measured using the wick-in-needle technique (Fadnes et al, 1977). A 23-gauge needle (Microlance, Dublin, Ireland), filled with multifilamentous nylon thread, was connected to an Abbott Transpac II pressure transducer (Abbott Ireland, Sligo, Ireland) by a polyethylene tubing filled with sterile heparinized (70 units $ml^{-1}$) saline. The nylon thread increased the contact area and improved the fluid communication in the needle. The pressure transducer was connected to a model 13–6615–50 preamplifier and a model TA240 Easygraf dual-channel chart recorder (Gould, Cleveland, OH, USA). The equipment was calibrated by determining the linear relationship between imposed pressure and measured pressure. The pressure of 30 cm of saline

**Figure 1** Proton $T_1$ (**A**) and $T_2$ (**B**) distributions of a typical R-18 human melanoma xenografted tumour was maintained for 5 min to test for possible leaks in the system. The needle was inserted in the central region of the tumour for measurement of IFP. The IFP was recorded for at least 10 min. After a stable IFP value was reached, the fluid communication between the pressure transducer and the tumour was tested by compressing and decompressing the tubing between the needle and the transducer using a screw clamp (Tufto and Rofstad, 1995). Measurements were discarded if the readings following these tests differed by more than 1 mmHg. Tumour IFP was determined by calculating the mean of these two readings. The IFP measured in normal tissue, i.e. subdermally in tumour-free dorsal skin or intramuscularly in the proximal portion of the lower extremity, served as an internal control.

### Statistical analysis

Statistically significant correlations were searched for by linear regression analysis. A significance criterion of $P < 0.05$ was used.

### RESULTS

Twenty-two R-18 tumours were subjected to investigation. The $T_1$ and $T_2$ distributions of a typical tumour are illustrated in Figure 1. The proton relaxation times were not uniform across the tumour; $T_1$ ranged from 1552 to 1788 ms and $T_2$ from 56 to 63 ms. Median $T_1$ and $T_2$ differed amomg individual tumours from 1379 to 1939 ms and from 58 to 77 ms respectively. The coefficient of variation

© Cancer Research Campaign 1997

**Figure 2** Proton $T_1$ (**A**) and $T_2$ (**B**) *vs* interstitial fluid pressure for R-18 human melanoma xenografted tumours. Points represent median values of individual tumours for a single tumour ranged from 3% to 14% for $T_1$ and from 2% to 7% for $T_2$. There was no correlation between median $T_1$ or $T_2$ and tumour volume ($P > 0.05$ for $T_1$ and $T_2$). Median $T_1$ and $T_2$ were not correlated with each other either ($P > 0.05$).

Fractional tumour water content covered a narrow range from 79% to 82%. Tumours with a high fractional water content showed a longer median $T_1$ than tumours with a low fractional water content. The relationship between median $T_1$ and fractional tumour water content was statistically significant ($P < 0.05$) despite the narrow range of the latter parameter. There was no correlation between median $T_2$ and fractional tumour water content ($P > 0.05$).

The IFP measured subdermally in tumour-free dorsal skin or intramuscularly in the proximal portion of the lower extremity ranged from –1 to +1 mmHg. On the other hand, the IFP was elevated in all tumours and covered a wide range from 5 to 31 mmHg. There was no correlation between IFP and tumour volume ($P > 0.05$). The IFP showed no correlation with fractional tumour water content either ($P > 0.05$). Moreover, there was no correlation between the proton relaxation times and IFP, irrespective of whether median $T_1$ or $T_2$ was considered ($P > 0.05$ for $T_1$ and $T_2$) (Figure 2).

### DISCUSSION

R-18 human melanoma xenografted tumours growing orthotopically in congenitally athymic nude mice were chosen as the model system in the present study. This model system is suitable for elucidating the question under consideration for several reasons. First, many essential biological properties of the donor patient's tumour, including angiogenic, vascular, histopathological and pathophysiological parameters, have been shown to be retained in R-18 tumours (Rofstad, 1994). Moreover, R-18 tumours are amelanotic, implying that paramagnetic relaxation enhancement by melanin is avoided (Atlas et al, 1990). Finally, the fraction of necrotic tissue in R-18 tumours is usually less than 3%, implying that MRI data are not confounded by necrosis-induced $T_1$ and $T_2$ shortening (Jakobsen et al, 1995).

Several methods for calculation of proton $T_1$ and $T_2$ values from MR images have been reported, and experiments with test phantoms have shown that the calculated values at best agree within about 10% of those measured by MR spectroscopy (Schneiders et al, 1983; Bakker et al, 1984). A relatively simple model was used to calculate $T_1$ and $T_2$ in the present work, ignoring the possibility that the relaxation process might be bi- or multiexponential. Moreover, correction for $T_2$ decay during signal aquisition was not included in the model. However, experiments with gadolinium diethylenetriamine penta-acetic acid (Gd-DTPA) phantoms have verified linear correlations between $1/T_1$ calculated from images and $1/T_1$ measured by relaxometry and between $1/T_2$ calculated from images and $1/T_2$ measured by relaxometry (Rofstad et al, 1994). Although the numeric values for $T_1$ and $T_2$ calculated here might deviate somewhat from the true values, our model gives accurate relative values for the relaxation times. Consequently, our values for $T_1$ and $T_2$ are valid for use in correlation analyses.

No correlation between median $T_1$ or $T_2$ and IFP was found for R-18 tumours. The lack of correlation can probably not be attributed to inadequate experimental procedures. MRI and IFP measurements according to the present procedures give highly reproducible results, as has been verified by performing repeated experiments with the same tumours (Rofstad et al, 1994; Tufto and Rofstad, 1995). The differences in $T_1$ and $T_2$ between tumours were larger than those between voxels in a single tumour, suggesting that the measured differences in median $T_1$ and $T_2$ are biological rather than methodological in origin. Moreover, the values for median $T_1$, median $T_2$ and IFP determined here are most probably representative for the whole tumour. Median $T_1$ and $T_2$ of a tumour were calculated from a single central axial scan. Single-scan and multiscan experiments have been shown to give almost identical median values for $T_1$ and $T_2$ in non-necrotic tumours (Rofstad et al, 1994). IFP was measured in a single location in the central region of a tumour. This procedure is justified by theoretical and experimental studies which have suggested that, in tumours growing as a single nodule, the IFP is relatively uniform throughout the tumour and drops precipitously to normal tissue values at the tumour–normal tissue interface (Jain and Baxter, 1988; Boucher et al, 1990).

Median $T_1$ was found to be positively correlated with fractional tumour water content, in agreement with the present hypothesis. However, a statistically significant correlation was not found between median $T_2$ and fractional tumour water content. It is probable that this does not mean that the proton $T_2$ values of R-18 tumours are not determined mainly by the water content of the tumour tissue. The lack of a significant correlation was rather a consequence of the small differences in fractional water content between individual tumours. This interpretation is supported by a previous observation; a strong correlation was found between median $T_2$ and fractional tumour water content when human melanoma xenografted tumours of different lines showing large

 © Cancer Research Campaign 1997

differences in fractional tumour water content were studied (Rofstad et al, 1994).

The range of distribution of IFP in R-18 tumours is sufficiently large that significant correlations between proton relaxation times and IFP should have been detected if present. The IFP of individual R-18 tumours ranged from 5 to 31 mmHg. This range of distribution is similar to those reported for experimental rodent and human tumours in general (Boucher et al, 1990; Zlotecki et al, 1993; Kristjansen et al, 1993; Znati et al, 1996) and melanomas in humans (Boucher et al, 1991; Curti et al, 1993).

The present study was based on the hypothesis that an increase in tumour IFP was accompanied by an increase in fractional tumour water content, which would be detected by MRI as an increase in proton $T_1$ and $T_2$ values. The IFP was found to differ significantly among individual tumours despite differences in the fractional tumour water content, and there was no correlation between these two parameters. The compliance of the tumour tissue, i.e. $C = \delta V/\delta P$ where $C$ is compliance, $\delta V$ is increase in fluid volume and $\delta P$ is increase in IFP, was probably not sufficiently large that the fractional tumour water content was influenced significantly by the IFP. Other tumour parameters, particularly parameters influencing the volume of the extracellular space such as the amount and distribution of stromal components and cellular adhesion molecules, were probably more determinative for the fractional tumour water content than the IFP.

The study reported here was performed using tumours of a single melanoma line, i.e. tumours that were of the same origin and thus were similar, and no correlation was found between proton $T_1$ or $T_2$ and IFP. Significant correlations are less likely to be found in studies involving tumours of different lines than in studies involving tumours of the same line (Rofstad, 1994). Experimental studies involving tumours of different lines are analogous to clinical studies. Consequently, the present study provides strong evidence against the hypothesis that proton $T_1$ and $T_2$ values determined by MRI can be used clinically to assess tumour IFP and thereby to predict the uptake of macromolecular therapeutic agents.

### ACKNOWLEDGEMENT

Financial support was received from The Norwegian Cancer Society.

### REFERENCES

Atlas SW, Braffman BH, Lobrutto R, Elder DE and Herlyn D (1990) Human malignant melanomas with varying degree of melanin content in nude mice: MR imaging, histopathology, and electron paramagnetic resonance. *J Comput Ass Tomogr* **14**: 547–554

Bakker CJG, De Graff CN and Van Dijk P (1984) Derivation of quantitative information in NMR imaging: a phantom study. *Phys Med Biol* **29**: 1511–1525

Belfi CA, Medendorp SV and NGO Fqh (1991) The response of the KHT sarcoma to radiotherapy as measured by water proton NMR relaxation times: relationships with tumor volume and water content. *Int J Radiat Oncol Biol Phys* **20**: 497–507

Boucher Y, Baxter LT and Jain RK (1990) Interstitial pressure gradients in tissue-isolated and subcutaneous tumors: implications for therapy. *Cancer Res* **50**: 4478–4484

Boucher Y, Kirkwood JM, Opacic D, Desantis M and Jain RK (1991) Interstitial hypertension in superficial metastatic melanomas in humans. *Cancer Res* **51**: 6691–6694

Boucher Y and Jain RK (1992) Microvascular pressure is the principal driving force for interstitial hypertension in solid tumours: implications for vascular collapse. *Cancer Res* **52**: 5110–5114

Bradwell AR, Fairweather DS, Dykes PW, Keeling A, Vaughan A and Taylor J (1985) Limiting factors in the localization of tumors with radiolabeled antibodies. *Immunol Today* **6**: 163–170

Braunschweiger PE, Schiffer LM and Furmanskip (1986) $^1$H NMR relaxation times and water compartmentalization in experimental tumor models. *Magn Reson Imaging* **4**: 335–342

Cobb LM (1989) Intratumour factors influencing the access of antibody to tumour cells. *Cancer Immunol Immunother* **28**: 235–240

Curti BD, Urba WJ, Alvord WG, Janik JE, Smith JW, Madara K and Longo DL (1993) Interstitial pressure of subcutaneous nodules in melanoma and lymphoma patients: changes during treatment. *Cancer Res* **53**: 2204–2207

Fadnes HO, Reed R and Aukland K (1977) Interstitial fluid pressure in rats measured with a modified wick technique. *Microvasc Res* **14**: 27–36

Jain RK (1987) Transport of molecules in the tumor interstitium: a review. *Cancer Res* **47**: 3039–3051

Jain RK and Baxter LT (1988) Mechanisms of heterogeneous distribution of monoclonal antibodies and other macromolecules in tumors: significance of elevated interstitial pressure. *Cancer Res* **48**: 7022–7032

Jakobsen I, Kaalhus O, Lyng H and Rofstad EK (1995) Detection of necrosis in human tumour xenografts by proton magnetic resonance imaging. *Br J Cancer* **71**: 456–461.

Jones PL, Gallagher BM and Sands H (1986) Autoradiographic analysis of monoclonal antibody distribution in human colon and breast tumor xenografts. *Cancer Immunol Immunother* **22**: 139–143

Kristjansen PEG, Boucher Y and Jain RK (1993) Dexamethasone reduces the interstitial fluid pressure in a human colon adenocarcinoma xenograft. *Cancer Res* **53**: 4764–4766

Lee I, Boucher Y and Jain RK (1992) Nicotinamide can lower tumor interstitial fluid pressure: mechanistic and therapeutic implications. *Cancer Res* **52**: 3237–3240

Less JR, Posner MC, Boucher Y, Borochovitz D, Wolmark N and Jain RK (1992) Interstitial hypertension in human breast and colorectal tumors. *Cancer Res* **52**: 6371–6374

Leunig M, Goetz AE, Gamarra F, Zetterer G, Messmer K and Jain RK (1994) Photodynamic therapy-induced alterations in interstitial fluid pressure, volume and water content of an amelanotic melanoma in the hamster. *Br J. Cancer* **69**: 101–103

Rofstad EK (1994) Orthotopic human melanoma xenograft model systems for studies of tumour angiogenesis, pathophysiology, treatment sensitivity and metastatic pattern. *Br J Cancer* **70**: 804–812

Rofstad EK, Steinsland E, Kaalhus O, Chang YB, Høvik B and Lyng H (1994) Magnetic resonance imaging of human melanoma xenografts *in vivo*: proton spin-lattice and spin-spin relaxation times versus fractional tumour water content and fraction of necrotic tumour tissue. *Int J Radiat Biol* **65**: 387–402

Sands H, Jones PL, Shah SA, Palme D, Vessella RL and Gallagher BM (1988) Correlation of vascular permeability and blood flow with monoclonal antibody uptake by human Clouser and renal cell xenografts. *Cancer Res*, **48**, 188–193

Schneiders NJ, Post H, Brunner P, Ford J, Bryan RN and Willcott MR (1983) Accurate $T_2$ images. *Med Phys* **10**: 642–645.

Steen RG (1992) Edema and tumor perfusion: characterization by quantitative $^1$H MR imaging. *Am J Roentgenol* **158**: 259–264.

Tufto I and Rofstad EK (1995) Interstitial fluid pressure in human melanoma xenografts. Relationship to fractional tumor water content, tumor size, and tumor volume-doubling time. *Acta Oncol* **34**: 361–365.

Zlotecki RA, Boucher Y, Lee I, Baxter LT and Jain RK (1993) Effect of angiotensin II induced hypertension on tumor blood flow and interstitial fluid pressure. *Cancer Res* **53**: 2466–2468.

Znati CA, Rosenstein M, Boucher Y, Epperly MW, Bloomer WD and Jain RK (1996) Effect of radiation on interstitial fluid pressure and oxygenation in a human tumor xenograft. *Cancer Res* **56**: 964–968

© Cancer Research Campaign 1997